# Preparing Sodium Alginate/Polyethyleneimine Spheres for Potential Application of Killing Tumor Cells by Reducing the Concentration of Copper Ions in the Lesions of Colon Cancer

**DOI:** 10.3390/ma12091570

**Published:** 2019-05-13

**Authors:** Ru Xu, Chen Su, Longlong Cui, Kun Zhang, Jingan Li

**Affiliations:** 1 School of Life Science, Zhengzhou University, Zhengzhou 450000, China; 18341349663@163.com (R.X.); suchenqwer@163.com (C.S.); whitebear24@163.com (L.C.); 2 School of Material Science and Engineering & Henan Key Laboratory of Advanced Magnesium Alloy & Key Laboratory of materials processing and mold technology (Ministry of Education), Zhengzhou University, Zhengzhou 450001, China

**Keywords:** colon cancer cells, copper ions, hydrogel sphere, sodium alginate, polyethyleneimine

## Abstract

Inhibition of residual malignant tumors in patients with colon cancer after operation is one of the difficulties in rehabilitation treatment. At present, using biocompatible materials to remove the copper ion which is the growth dependence of malignant tumors in the lesion site is considered to be the frontier means to solve this problem. In this work, we developed a sodium alginate (SA)/polyethyleneimine (PEI) hydrogel sphere via cross-linking method (SA/SP/SA; SP = SA/PEI) as an oral biomaterial for adsorbing and removing copper ions from colon cancer lesions. The evaluated results showed that the SA/PEI/SA (SPS) hydrogel sphere obtained the largest swelling rate at pH 8.3 which was the acid-base value of colon microenvironment and absorbed more copper ions compared with the SA control. The cell experiment presented that the SPS hydrogel sphere owned better compatibility on normal fibroblasts and promoted higher death of colon cancer cells compared with SA/PEI (SP) and SA control. Our data suggested that the SA/PEI hydrogel sphere had the potentiality as an oral biomaterial for inhibiting colon cancer cells.

## 1. Introduction

Colon cancer is a kind of malignant gastrointestinal cancer with a high incidence among people aged 40–50 years; it is the third most common cancer of that age group [1,2]. The main treatment method for colon cancer is surgical resection, supplemented by chemotherapy and drug treatment [3,4]. However, about half of patients may suffer from postoperative metastasis and recurrence that endanger their life [5]. In addition, some patients show no significant improvement on preoperative and postoperative radiotherapy [6]. Thus, there is an urgent need for a targeted and effective method to inhibit and kill colon cancer cells as an adjuvant or alternative therapy for surgery and chemotherapy. Studies have shown that the accumulation of copper ions in cancerous sites is an important factor for the survival and proliferation of cancer cells [7,8]; hence, removal of copper ions accumulated in the colon may be effective in killing cancer cells.

The technical bottleneck, then, is how to remove the copper ions from the focal site without damaging healthy cells. Hydrogels with good swelling properties are undoubtedly preferred, and preparing the hydrogels as oral biomaterials (e.g., hydrogel spheres) is a good adjuvant therapy for colon cancer patients after operation. However, the oral hydrogel spheres will go through different organs of the digestive system, such as stomach (pH 1.2), rectum (pH 6.8), blood (pH 7.4) and colon (pH 8.3), the pH microenvironment of which will affect the swelling properties of the materials [9,10]. Thus, choosing a hydrogel material with maximum swelling rate in colon microenvironment is the key to solve this problem. Sodium alginate (SA) hydrogel is a kind of pH-sensitive hydrogel, but its swelling rate is stable above pH 7.4 [11]. We also found that SA hydrogel has good plasticity and biocompatibility in our previous work [12]. However, the porous structures of tens of micron scales on the surface of SA hydrogels may not be conducive to the implementation of this function: The porous connectivity structure of hydrogels may cause the exudation of inhaled copper ions and reduce the efficiency of carrying copper [13]; on the other hand, pores with a diameter of tens of micron-scales may allow most cells to migrate and aggregate, and the migrated cells will cause long-term hydrogel retention in the body, delaying the excretion of adsorbed copper ions outside [14]. In the previous research, polyethyleneimine (PEI) molecules were modified on the materials surface via layer-by-layer self-assembly and were proved to inhibit series of pathologically related cells of esophageal cancer [15,16]. Thus, modifying the SA hydrogel with PEI and SA molecules may improve its surface compactness and swelling degree, further endowing the hydrogel function of inhibiting cancer cells.

In this contribution, the SA/PEI/SA (SPS) hydrogel sphere was prepared via the cross-linking method aimed at absorbing and removing the copper ion and further killing the colon cancer cell. The SPS hydrogel sphere’s surface morphology, inner structure, swelling ratio, and ability on removing copper ion and promoting colon cancer cell apoptosis were investigated systematically. We hope this SPS hydrogel sphere may have potential application as an oral biomaterial for postoperative rehabilitation of patients with colon cancer.

## 2. Materials and Methods 

### 2.1. Preparation of Sodium Alginate/Polyvinylimide Hydrogel Spheres via Crosslinking

**Figure 1:**
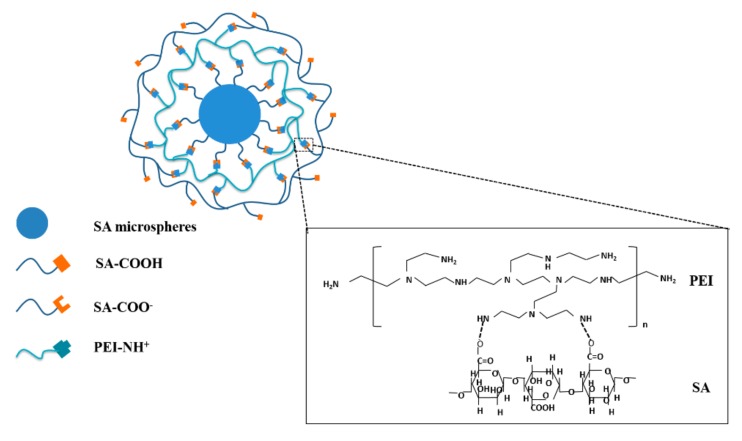
Preparation of SA/PEI/SA (SPS) spheres. SA: sodium alginate; PEI: polyethyleneimine.

Sodium alginate (SA, 3.2 × 10^4^ Da–2.5 × 10^5^ Da, Sigma) was dissolved in deionized water (dH_2_O) to prepare a solution of 20 mg/mL. Thereafter, the SA solution was added to 100 mg/mL calcium chloride (CaCl_2_) supersaturated solution (dissolved in the dH_2_O) drop by drop using an injector with 1 mL capacity, and followed with 30 min magnetic stirring. After filtering residual solution and cleaning, SA hydrogel spheres that would be placed in the core position were obtained. Next, the SA hydrogel spheres were poured into polyethyleneimine (PEI, 2.5 × 10^4^ Da, Sigma) solution (20 mg/mL, dissolved in the dH_2_O) and stirred for 30 min. After filtering residual solution and clear operation once more, the obtained samples were SA spheres covered with a layer of PEI molecule and were labeled as SP. Then the SP spheres were put into 20 mg/mL SA solution again and stirred for another 30 min. By the final filtering of residual solution and cleaning step, the acquired samples were SP spheres overlapped with another layer of SA and were named as SPS. The preparation process of SPS hydrogel spheres is exhibited in Figure 1.

### 2.2. Characterization of the SPS Hydrogel Sphere

The infrared absorption spectra of the SPS, SP and SA samples were gained using a Fourier transform infrared (FTIR, NICOLET 5700, Waltham, MA, USA) spectrometer in the scanning range of 4000–400 cm^−1^ after being freeze-dried, crushed, ground and tablet compressed with KBr [17]. The SPS, SP and SA hydrogel spheres were photographed with Huawei Mate 10 Pro camera equipment to directly observe their entire object and sizes, and their diameters were also detected from at least 100 spheres for each sample [18]. Surface and cross-section morphologies as well as surface energy dispersive spectrum (EDS) of the SA, SP and SPS hydrogel spheres were observed by scanning electron microscopy (SEM, FEI Quanta200, The Netherlands) after freezing at −80 °C, being fully dried with a freeze-dryer, and gold spraying [19].

The swelling ratios (SR) of the SPS hydrogel sphere within 18 h were determined and calculated in the following formula: SR = m1 − m0/m0, wherein m0 indicated the dry weight of the samples and m1 indicated the wet weight of the samples [12]. In addition, the SR values and degradation ratio of the SPS under condition of pH 1.2, pH 6.8, pH 7.4 and pH 8.3 were detected for the consideration of the pH difference in vivo microenvironment that the oral colon cancer drugs may go through: The pH value of stomach is 1.2, the pH value of rectum is 6.8, the pH value of blood is 7.4 and the pH value of colon is 8.3 [20,21]. The absorption ratios of the SA, SP and SPS samples after exposure to different cumulative pH (pH 1.2 for 4 h, pH 7.4 for 8 h, and pH 8.3 for 60 h) were also investigated.

To investigate the ability of SPS, SP and SA hydrogels spheres on absorbing copper ion we performed the experiment as follow [22]: The concentrations of copper sulphate solution (dissolved in dH_2_O) were designed as 22.0 mg/L, with pH 8.3. The SA, SP and SPS spheres (200 mg per group) were immersed in the copper sulfate solution separately, and the solution with spheres were magnetic stirred by 150 rpm at 37 °C. Two mL solutions were taken out from each group after 1 h, 2 h, 3 h, 4 h, 5 h, 6 h, 18 h and 24 h to determine their absorbance by ultraviolet spectrophotometer, and the concentration of copper ion in solution was determined according to the standard curve.

### 2.3. Biocompatibility Testing of the Developed Hydrogels Spheres

A first set of experiments was performed to evaluate in vitro biocompatibility of the SPS, SP and SA hydrogel spheres by using L929 cell line. These fibroblast-like cells were seeded at an initial cell density of 4000 cells/well and incubated at 37 °C in culture medium (90% Dulbecco’s Modified Eagle medium high glucose, 10% fetal bovine serum, 100 units/mL penicillin and 0.1 mg/mL streptomycin solution) until they reached 60% confluence. At this time point, the cells were treated with SPS, SP and SA hydrogel spheres (4 spheres per well) for 1 day, 2 days and 3 days. For comparative purposes, the cells cultured with no hydrogels spheres were also investigated and considered as control (CON) group. After each incubation period the cells were stained with the cell-permeable acridine orange (AO) in combination with the plasma membrane-impermeable DNA-binding dye propidium iodide (PI). AO and PI excite green and red fluorescence, respectively, when they are intercalated into the cells’ DNA, which represents living or dead cells, because only AO was able to cross the plasma membrane of living cells, while PI can only cross the plasma membrane of dead or dying cells [23]. L929 survival rates in each group were counted and calculated from at least 15 images [15].

### 2.4. Investigation of the Hydrogel Spheres’ Capacity to Kill Colon Cancer Cells

Human colon cancer cells (HT-29, Shanghai Zhong Qiao Xin Zhou Biotechnology Co., Ltd., Shanghai, China) were seeded in the 24-well cell culture plate with a density of 4000 cells/well, and incubated in a humidified incubator with 95% air and 5% CO_2_ at 37 °C. When the cells reach 60% of the confluence, the SPS hydrogel spheres prepared under aseptic conditions were placed in the culture plate in a density of 4 per well, and co-cultured with the colon cancer cells for 1 day, 2 days and 3 days, respectively [24]. The SP, SA and non-hydrogel sphere co-cultured cells (the group was labeled as CON) were also incubated as comparison. The function of SPS on suppressing colon cancer cells was also evaluated by staining with AO/PI. The inhibition rate of each group on colon cancer cells was also counted from the fluorescence images [15].

The study was conducted in accordance with the Declaration of Helsinki, and the protocol was approved by the Ethics Committee of Zhengzhou University.

## 3. Results and discussion

### 3.1. Physical and Chemical Properties of the SPS Hydrogel Sphere

**Figure 2:**
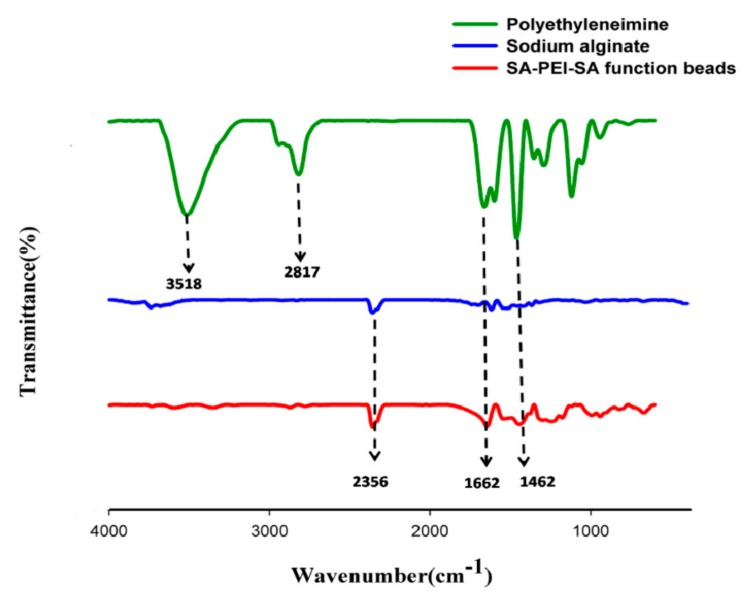
Fourier transform infrared (FTIR) spectra of PEI, SA and SPS samples.

The chemical structures of SPS sample and the SA and PEI controls were analyzed by FTIR spectroscopy, and are presented in Figure 2: The absorption peaks of 1672 cm^−1^ in SPS spheres came from the bending vibration peaks of N-H groups in PEI, and there were obvious double absorption peaks at 3700–3100 cm^−1^, which were obvious amino characteristic peaks; the hydroxyl absorption peaks in the SA curve disappeared in the SPS spheres, which may be due to the destruction of hydroxyl groups by Ca^2+^ ion bonding; in SPS spheres, 2648–3030 cm^−1^ was the stretching and bending vibration peaks of CH_2_ group in PEI molecule and 1143–1359 cm^−1^ was the stretching and retracting absorption peaks of carboxyl group in SA molecule; SA and PEI participated in the reaction by forming amide bond, so the peak value of N-H (3500–3400cm^−1^) single bond of PEI molecule in SPS decreased; at the same time, due to the spatial change of N-H bonds in the reaction, the absorption peaks of N-H bonds shifted; in SPS, the characteristic peaks of amide bond and the absorption peaks of amide I bond C=O (1690–1630 cm^−1^) were enhanced. Thus, it could be summarized that SPS spheres were bound together by carboxyl and amino groups to form amide bonds.

**Figure 3:**
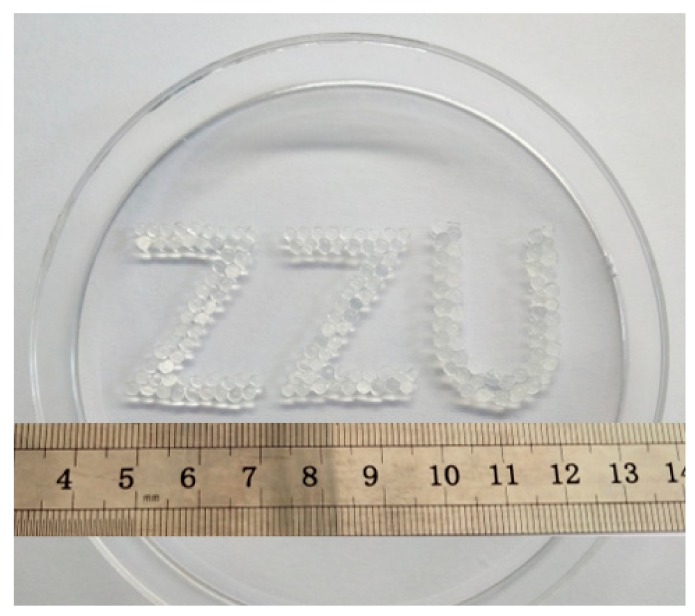
Photographs of the abbreviation of Zhengzhou University, “ZZU”, which was composed of the SA (the first letter “Z”), SA/PEI (SP) (the second letter “Z”) and SPS (the third letter “U”) hydrogel spheres.

Figure 3 depicts a design of the abbreviation of our institution Zhengzhou University, “ZZU”, which was composed of three kinds of hydrogel spheres, SA, SP and SPS: From left to right, the first Z is made of SA balls, the second Z is made of SP balls and the U is made of SPS balls. It could be seen from the figure that the sizes of SA and SP spheres were relatively the same, and they were transparent. SPS spheres were larger than the first two kinds of spheres and were light yellow. Because the color of sodium alginate solution was light yellow, the spheres encapsulated with sodium alginate showed the same color. Statistical results of the sphere diameters in Table 1 further verified the visual images, displaying their trend of SPS > SP > SA.

**Figure 4:**
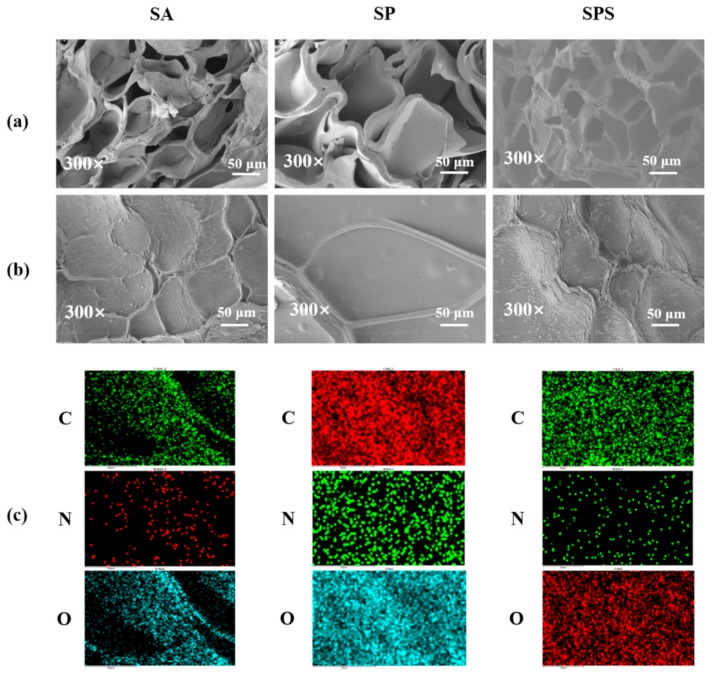
The (**a**) cross-section, (**b**) surface microstructure and (**c**) surface energy dispersive spectrum (EDS) of the SA, SP and SPS hydrogel sphere observed by scanning electron microscopy (SEM).

After freeze-drying of SA, SP and SPS hydrogel and breaking by liquid nitrogen, the micro-morphologies and internal structure of its surface and cross section was observed. The cross-sectional graph of SA, SP and SPS exhibits obvious three-dimensional network cross-linking structure and porous morphology (Figure 4a), and this is a typical hydrogel inner structure which contributed to better swelling property and further absorbing more copper ion from the lesion location [25]. Wherein, SA and SP show larger pore diameters and thicker pore walls compared with SPS. The surface morphology of the SA, SP and SPS hydrogel sphere is compact in the micro sizes and there are obvious wrinkles (Figure 4b), which is completely different from the morphology of the existing porous, coherent and open hydrogels [26]. The latter, as scaffolds for tissue engineering, often promotes cell migration, proliferation and differentiation [27], while the former, as a targeted anti-tumor biomaterial, should inhibit cell growth to avoid prolonged retention of SPS spheres and their absorbed copper ion in vivo. Figure 4c displays the content distribution of C, O and N elements in the surface analysis of each sample: The surface of SA spheres contains a lot of C and O elements, but a very small amount of N elements also appears on the surface, possibly due to a false positive result; in addition to a large number of C and O elements, N elements on the surface of SP spheres increase significantly compared with SA spheres, indicating that the PEI molecule has been successfully encapsulated on the surface of SA spheres; the surface composition of SPS spheres is similar to that of SA spheres, indicating that the sodium alginate solution successfully wraps the PEI on the surface of SP spheres.

**Figure 5:**
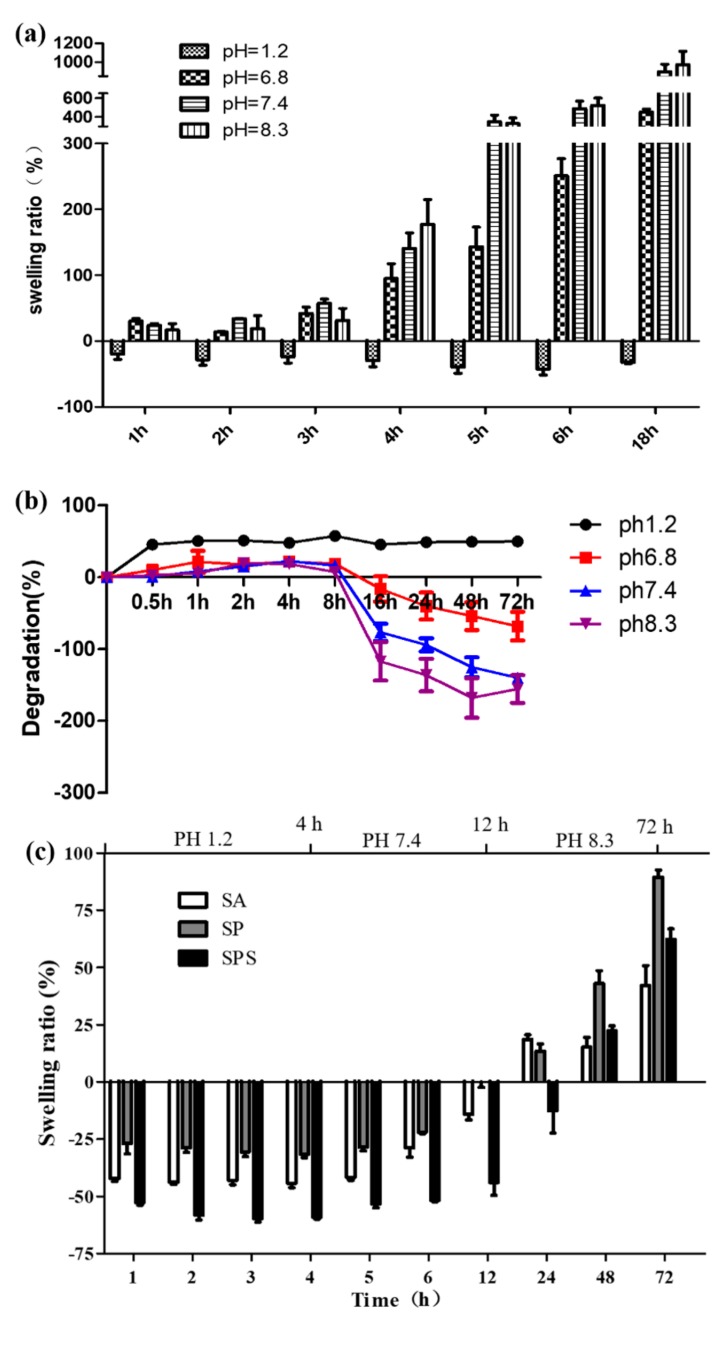
(**a**) The swelling ratio of the SPS hydrogel sphere after immersed in the phosphate buffer solution for 1 h, 2 h, 3 h, 4 h, 5 h, 6 h and 18 h in the condition of pH 1.2, pH 6.8, pH 7.4 and pH 8.3, separately; (**b**) the degradation ratio of the SPS hydrogel sphere for 0.5 h, 1 h, 2 h, 4 h, 8 h, 16 h, 24 h, 48 h and 72 h in the condition of pH 1.2, pH 6.8, pH 7.4 and pH 8.3, separately; (**c**) the swelling ratio of the SA, SP and SPS hydrogel spheres after immersion in the PBS solution in the condition of pH 1.2 for 4 h, and then pH 7.4 for 8 h, and finally pH 8.3 for 60 h. (mean ± SD, n = 5).

Oral colon cancer medicines pass through the stomach and rectum before reaching the site of the lesion, where the pH environment is quite different from that of the colon [16,28,29]. In this research, we measured the SR values of the SPS hydrogel sphere under different pH values according to the corresponding microenvironments over time (Figure 5a). The results show that the SR values of SPS hydrogel sphere increase with time in colon (pH = 8.3), rectum (pH = 6.8) and blood (pH = 7.4). From the 4th hour, the SR values of SPS hydrogel sphere in colon and blood microenvironment are significantly higher than that of other groups, and reach 586.3% at the 6th hour (pH = 8.3). These results indicate that SPS spheres have good swelling properties in intestinal environment (including blood microenvironment of wound after operation), and the increase of sphere volume is beneficial to its better adsorption of copper ions in vivo. In simulated gastric solution (pH = 1.2), the SR value of SPS spheres is much lower than that of other microenvironments, and there is no significant change over time. This is mainly due to the acidic environment that makes the carboxylic group of sodium alginate protonated, and the gel mesh is tightly adsorbed by the interaction of intermolecular electrostatic forces and hydrogen bonds. The degradation ratio result (Figure 5b) showed that pH value was a crucial factor which affects the SPS degradation behaviors. Under pH 1.2 condition, SPS maintains a stable degradation ratio of about 60%; while under other pH values, the SPS shows very low degradation ratio within the first 8 h; after 16 h at pH 6.8, 7.4 and 8.3, the spheres show a swelling trend, which is correlated with pH; the higher the pH value, the more obvious the swelling is; however, after 16 h at pH 1.2, the volume of the SPS spheres remain stable, and there is no obvious phenomenon of sharp increase or decrease; it can be inferred that the spheres are wrinkled under the influence of pH in the gastric environment and gradually show a swelling trend in the intestinal fluid environment. The absorption ratios of the SA, SP and SPS samples after exposure to different cumulative pH (Figure 5c) show that SA, SP and SPS spheres present different degrees of water loss in pH 1.2 environment; during the period of pH 7.4, the spheres begin to slowly absorb water and recover their original state; in the pH 8.3 environment, the spheres begin to swell, and the SP and SPS spheres possess higher swelling ratios compared with SA, wherein the swelling effect of SP spheres is the most obvious.

**Figure 6:**
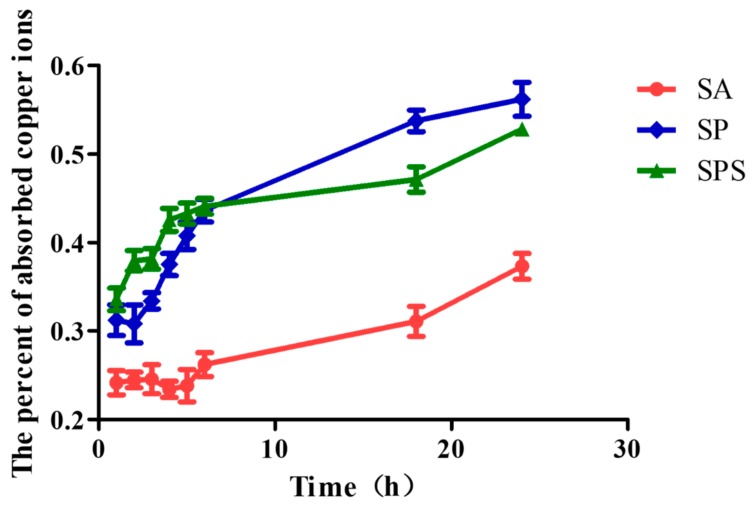
The ability of the SPS, SP and SA hydrogel spheres to absorb copper ions at pH 8.3 (mean ± SD, n = 5).

Figure 6 displays the percent of the absorbed copper ions of SPS, SP and SA hydrogel sphere within 24 h. It is obvious that SPS and SP samples absorb more copper ions compared with the SA sample which may be attributed to the good complexation ability of PEI with copper ions and the compact microstructures of the spheres. In addition, the SPS sample presents rapider absorption compared to SP within the first 6 h, and this will make more contribution to killing and inhibiting colon cancer cells, because the hydrogel sphere may be deferred for only several hours in the colon and then be excreted in vitro through feces.

### 3.2. Hydrogel Spheres’ Cytotoxicity against Normal Cells

**Figure 7:**
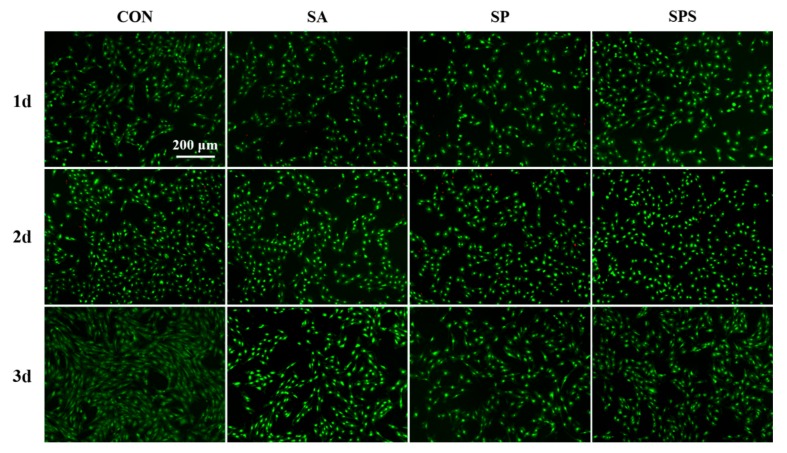
Acridine orange (AO) and propidium iodide (PI) staining images of L929 cell in the SPS, SP, SA and control (CON) groups. (The green dots stained by AO indicate living cells, and the red dots stained by PI indicate dead or dying cells).

**Figure 8:**
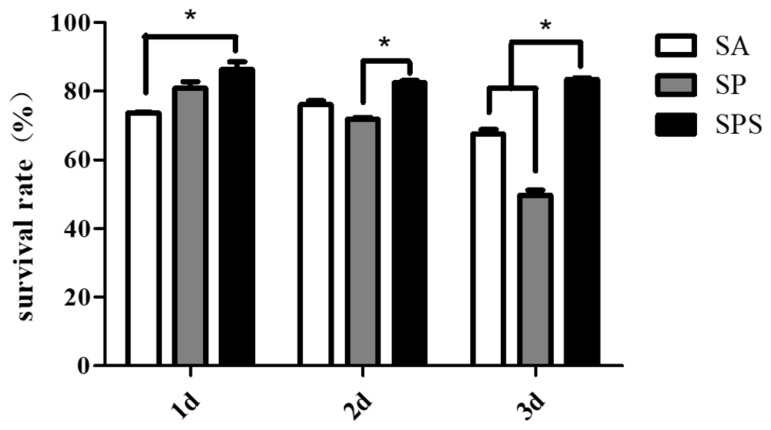
Inhibition rate of the SPS, SP and SA hydrogel spheres on the L929 cells (* *p* < 0.05, mean ± SD, n = 3).

To investigate the biocompatibility of the SPS hydrogel spheres, the SPS, SP and SA samples were co-cultured with the L929 cell line (Fibroblasts). The live/dead staining images (Figure 7) and the following cell counting results (Figure 8) indicate that the cells treated with SPS hydrogel spheres showed higher cell survival rates than those treated with SA and SP spheres. This finding suggests that the SPS spheres are the most biocompatible and well-tolerated by normal cells.

### 3.3. The Ability of SPS Hydrogel Spheres to Kill Colon Cancer Cells

**Figure 9:**
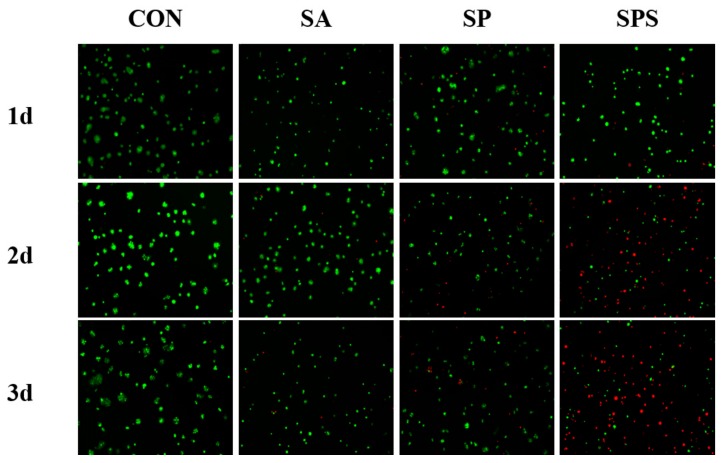
AO and PI staining images of colon cancer cells in the SPS, SP, SA and CON groups. (The green dots stained by AO indicate living cells, and the red dots stained by PI indicate dead or dying cells).

Pathological metastasis and proliferation of colon cancer cells after tissue resection is an important cause of recurrence of the disease [30]. Therefore, effective growth inhibition or killing of the colon cancer cells represent the main objective of researchers involved in the development of related drugs and biomaterials. Figure 9 presents the AO/PI staining images of colon cancer cells that co-cultured with the SPS, SP and SA hydrogel spheres, and the cancer cells cultured alone were also stained as control (the CON group). Wherein, the green fluorescence represented the living cells and the red fluorescence represent dead or dying cells. The SPS group shows obviously more red-labeled cells compared to other groups: At the 2nd day more than half of the colon cancer cells in the SPS group show red color, and at the 3rd day almost all the colon cancer cells present red color; only a few cells maintain green; this phenomenon indicates that the SPS hydrogel sphere had a strong function on inhibiting or killing the colon cancer cells.

**Figure 10:**
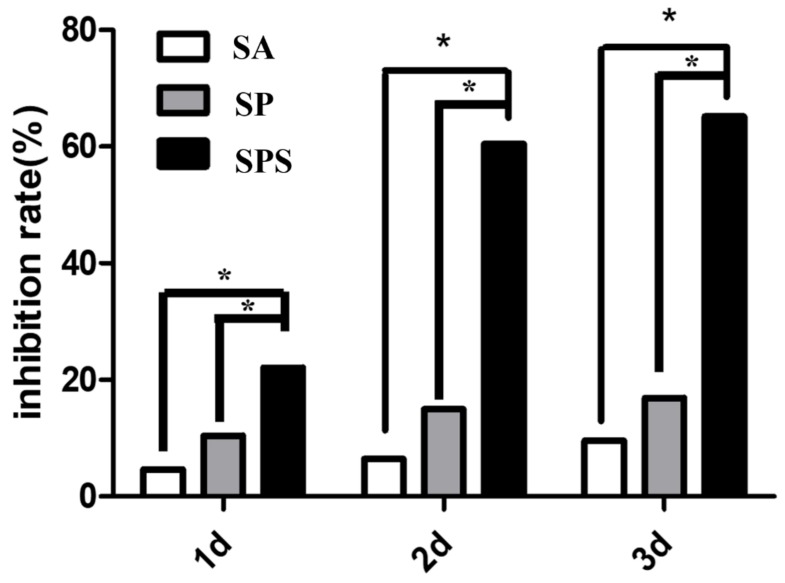
Inhibition rate of the SPS, SP and SA hydrogel spheres on the colon cancer cells (* *p* < 0.05).

The statistical results are consistent with the trend of AO/PI images (Figure 10). While the SA and SP group also exhibit certain properties on inhibiting the colon cancer cells, their inhibition rates are significantly lower compared with the SPS group’s value, and the trend is: SA < SP < SPS. The reason may be the reduced copper ions and the existing free carboxyl group [7,19]: SA has a certain amount of free carboxyl group, while SP reduces the copper ions; judging from the inhibition rate, reducing copper ions plays a more important role for inhibiting colon cancer cells; SPS has both the reduced copper ions and the free carboxyl group, thus showing the best function on killing colon cancer cells.

## 4. Conclusions

In this contribution, we developed an SPS hydrogel sphere as an adjuvant or alternative therapeutic material for post-operative radiotherapy of colon cancer. The aim of designing the SPS hydrogel was to reduce the aggregated copper ion from colon cancer lesions, which was considered as the dependence of the colon cancer cells pathological metastasis and hyperplasia. The SPS possessed an internal structure of porous networks, but a dense surface structure, which might endow it with a better swelling property, contributing to absorbing more copper ion from the focus. However, the SPS also had dense surface morphology at micron scale, and this would inhibit the cell growth into the sphere and avoid the retention of loaded copper ions in vivo. As an oral target biomaterial, the SPS had a lower swelling ratio in the gastric juice pH environment but a significantly higher swelling ratio in the intestinal pH environment. As we expected, compared with other hydrogel spheres, the SPS absorbed and removed more copper ions and further inhibited and killed colon cancer cells more effectively.

## Figures and Tables

**Table 1:** Diameter statistics of the SA, SP and SPS hydrogel spheres (n = 100, mean ± SD).

Samples	SA	SP	SPS
Diameters	2.4 ± 0.0 mm	2.5 ± 0.0 mm	2.7 ± 0.1 mm
